# Electrocardiogram Electrode Positioning on the Back During Echocardiography: An Exploratory Cross-Sectional Study

**DOI:** 10.7759/cureus.57967

**Published:** 2024-04-10

**Authors:** Satoshi Yamashita, Takeji Saitoh, Keisuke Iguchi, Kenichiro Suwa, Hayato Ohtani, Masao Saotome, Yuichiro Maekawa

**Affiliations:** 1 Department of Cardiology, Hamamatsu University School of Medicine, Hamamatsu, JPN; 2 Next Generation Creative Education Center for Medicine, Engineering, and Informatics, Hamamatsu University School of Medicine, Hamamatsu, JPN

**Keywords:** baseline fluctuation, breathing, electrocardiogram, body position, electrocardiogram electrodes

## Abstract

Background: ECG interpretation is sometimes difficult due to baseline fluctuations and electrode detachments when placed on the subjects’ front side, leading to misinterpretation of the rhythms and phases of the cardiac cycle. We aimed to compare the differences in the wave amplitudes and respiratory variations between conventional ECG electrode positioning on the front side of patients and an alternative position on the backs of patients.

Methods: Echocardiography was performed in 85 patients lying in the left lateral position. We attached the red electrode to the right clavicle, the yellow to the left clavicle, and the green to the left lateral abdomen on the front side of the patients; on the back, we attached the electrode to the right clavicle, the right upper posterior iliac spine, and the left upper posterior iliac spine.

Results: The ECG monitor amplitudes were greater on the front side compared to the back side, but the BF-breath values were smaller on the back side (6.0 pixels) compared to the front side (10.5 pixels, p<0.05). The P wave amplitude divided by the BF-breath on the back side was greater than that seen on the front side (2.8 vs. 1.8, p<0.05), whereas the QRS amplitude divided by the BF-breath was 15.0 and 16.3, respectively (p=ns).

Conclusion: As an alternative to front-side ECG monitoring, electrodes placed on the back can help avoid misinterpretation of the ECG rhythms and the phases of the cardiac cycle due to respiration during echocardiography.

## Introduction

Echocardiography is a non-invasive test that is essential in modern medicine, and it is the most widely used cardiac technology [1]. When performing echocardiography, it is important to attach the electrodes and view the results on the electrocardiogram monitor in order to detect the rhythms, view the E- and A-waves, and measure the left atrial and ventricular dimensions [2]. Originally, the electrocardiogram was based on Einthoven's triangular principle, which was established in 1905 and is the most widely used worldwide [3]. Three electrodes attached to an ECG monitor are used according to this principle. In particular, P waves can be observed in lead II, making it easy to detect rhythm [4,5]. However, the electrode positions of ECG devices used for echocardiography have not been clearly defined and vary depending on the examiner performing the test. Subsequently, most clinicians seem to place the electrodes on the front side of patients for echocardiography. In addition, the baseline fluctuation of the ECG monitor readings during patient breathing (BF-breath), which happens sometimes when we attach the electrodes on the front side, makes it difficult to interpret the rhythms.

The aim of this study was to investigate the differences in the wave amplitudes and respiratory changes between the conventional ECG electrode positioning on the front side of patients and a new ECG electrode position on patients’ backs.

## Materials and methods

Study population

This was an exploratory cross-sectional study conducted between December 15, 2018, and June 27, 2019, at Hamamatsu University Hospital, Hamamatsu, Japan. We performed echocardiography (Epiq7; Koninklijke Philips N.V., Amsterdam, Netherlands) on 100 consecutive patients, excluding 15 patients with atrial fibrillation and flutter. A total of 85 patients were enrolled in the study.

Procedure

**Figure 1:**
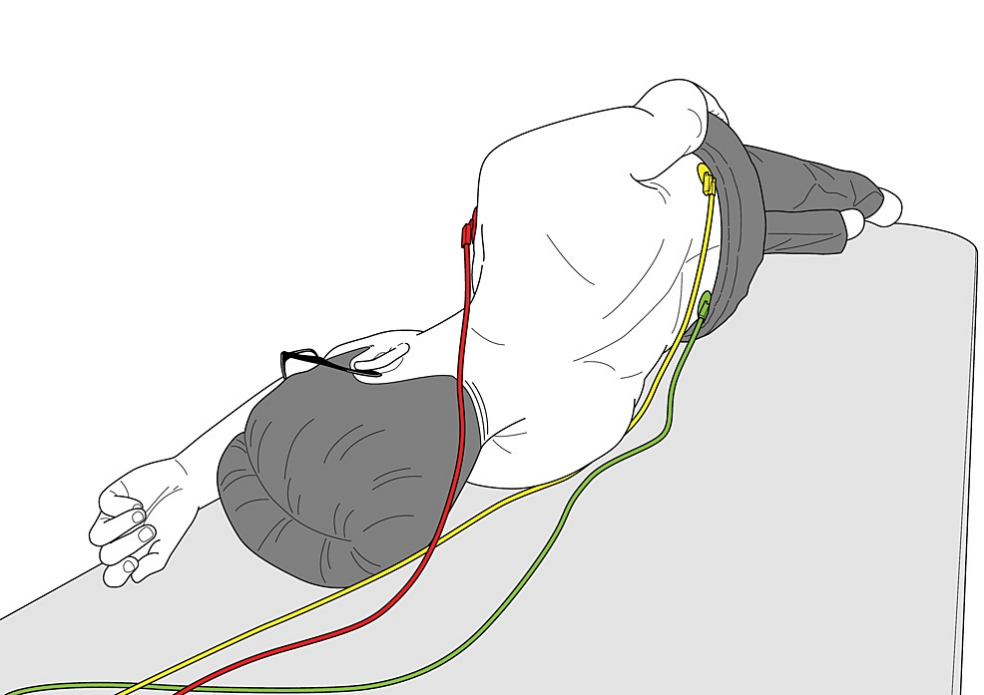
Attachment of the electrodes on the back side Image Credit: Authors

The test was performed with the patients in the left lateral decubitus position. We attached the red electrode to the right clavicle, the yellow to the left clavicle, and the green to the left lateral abdomen on the front side; on the back, the electrodes were attached to the right clavicle, the right upper posterior iliac spine, and the left upper posterior iliac spine of each patient, as shown in Figure 1.

Comparison of the ECG Measurements Between the Front-Side and Back-Side Electrode Positions

**Figure 2:**
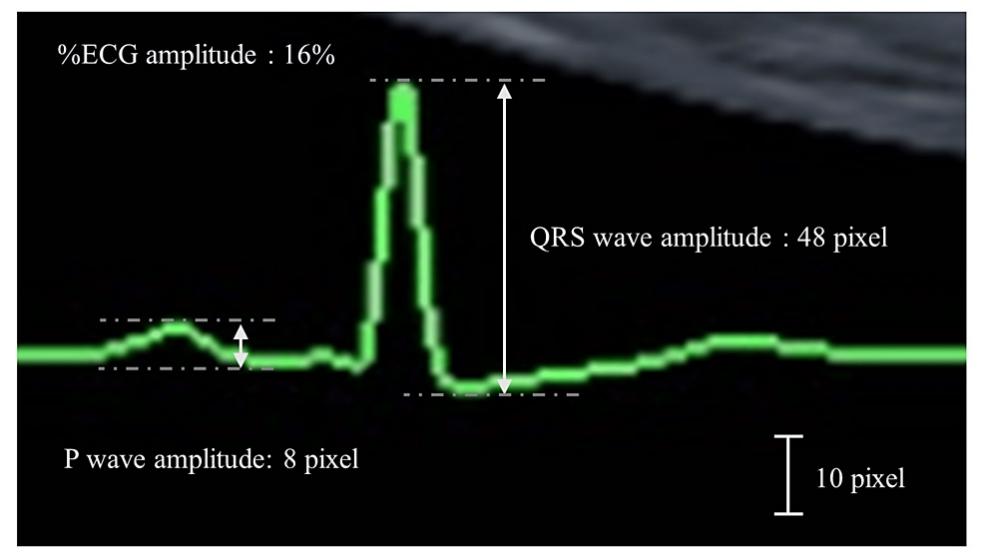
Monitoring electrocardiogram data in echocardiography Each P and QRS amplitude  (the shorter two-headed arrow and the longer two-headed arrow) was 8 and 48 pixels, respectively, at an amplitude of 16%. We calculated the values under an ECG amplitude of 100%. The amplitudes of the P and QRS waves were calculated and determined to be 50 pixels (= 8 × 100/16) and 300 pixels (= 48 × 100/16), respectively.

We set the %ECG amplitude on the monitor so that the QRS wave was displayed in the vertical width of the monitor, and then measured the P and QRS wave heights as pixels, starting from the leading edge and ending at the trailing edge (Figure 2). We then converted these heights to their original amplitude percentages of 100%, as presented in Figure 2.

**Figure 3:**
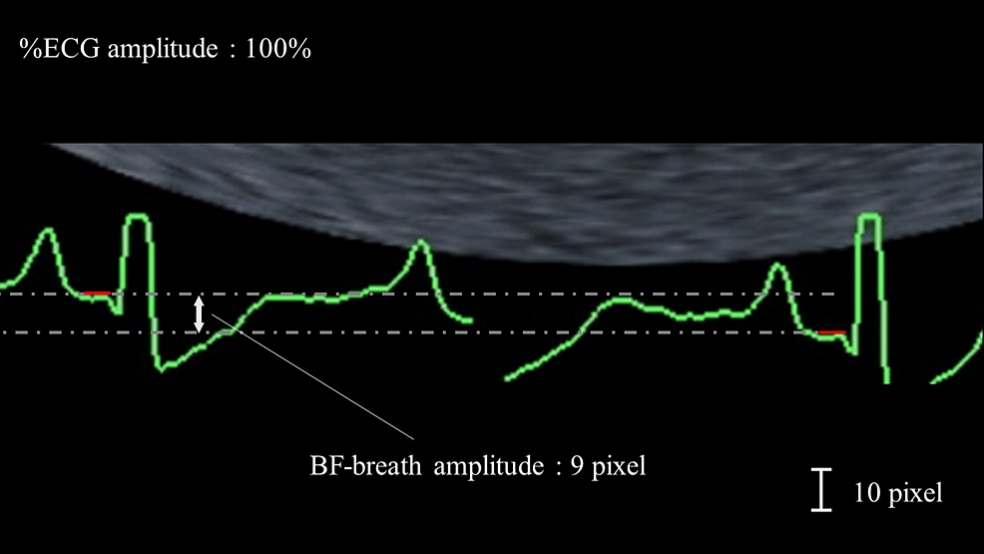
ECG baseline fluctuations during breathing BF-breath amplitude indicates the distance between the short red lines. BF-breath amplitude here is 9 pixels under 100% of %ECG amplitude. ECG, electrocardiogram; BF-breath, baseline fluctuation of the ECG monitor readings during patient breathing.

When evaluating BF-breath, we measured the distance between the highest and the lowest baselines on the monitor under an ECG amplitude of 100%, as shown in Figure 3. In addition, the P and QRS wave values were divided according to the BF-breath pixels to account for the influence of the breaths on the waveforms. We compared the ECG values obtained from the red-green leads between the front side and the back side, which are almost the same as lead II.

Statistical analysis

Continuous data were expressed as mean ± standard deviation (SD) when the data distribution was normal or as median with interquartile range (IQR) when the data was nonnormally distributed. Categorical data were expressed as numbers and percentages. Continuous variables were compared using the Wilcoxon rank sum test; p-values <0.05 were considered statistically significant. Associations between patient characteristics and BF-breaths were determined using Spearman’s rank correlation. All statistical analyses were performed using EZR (Easy R) (Saitama Medical Center, Jichi Medical University, Saitama, Japan), which is a graphical user interface for R (R Foundation for Statistical Computing, Vienna, Austria, version 3.3.2).

Ethical considerations

The study was approved by the Hamamatsu University Review Board (approval number: #18-166). All procedures followed were in accordance with the ethical standards of the responsible committee on human experimentation (Hamamatsu University Ethics Committee) and with the Helsinki Declaration of 1964 and later versions. Informed consent was obtained from all participants.

## Results

Patient characteristics

We performed echocardiography on 71 men and 39 women. Participants had a mean age of 68±13 years, height of 161.0±8.6 cm, weight of 59.7±12.5 kg, body surface area of 1.61±0.19 m^2^, and body mass index of 23.0±4.1 kg/m^2^. Sinus rhythm was present in 85% of the participants.

ECG monitoring and BF-breath

The amplitudes of the median P waves and QRS waves were greater on the front side compared to the back side electrode positions, as shown in Table 1, whereas the effects due to BF-breath were weaker when electrodes were on the back side compared to the front side.

**Table 1:** Wave amplitudes Data are shown as median (interquartile range). BF-breath, baseline fluctuation of the ECG monitor readings during patient breathing.

N = 85	Front side	Back side	P value
P, pixel	26.0 (20.0-34.0)	20.0 (15.0-30.0)	< 0.05
QRS, pixel	161.5 (103.5-237.8)	97.0 (73.0-142.3)	< 0.05
BF-breath, pixel	10.5 (6.0-18.5)	6.0 (2.8-10.0)	< 0.05

There was no strong relationship (r=0.04, -0.09, -0.16, -0.17, and -0.19) seen between BF-breath values in terms of amplitudes and the patients’ mean age, height, weight, BSA, and BMI, respectively, when the electrodes were placed on the back side (red-green).

The P wave amplitudes divided by the BF-breath values on the back side had greater values than those seen on the front side, as shown in Table 2 (p<0.05), while the QRS amplitudes divided by the BF-breath values showed no significant differences between the front side and the back side electrode positions.

**Table 2:** Wave amplitudes divided by BF-breaths Data are shown as median (interquartile range). BF-breath, baseline fluctuation of the ECG monitor readings during patient breathing.

N = 85	Front side	Back side	P value
P/BF-breath	1.8 (0.5-4.1)	2.8 (1.2-5.4)	< 0.05
QRS/BF-breath	15.0 (6.3-25.3)	16.3 (9.6-27.2)	ns

## Discussion

We investigated the values measured on the ECG monitor when electrodes were placed on the back of patients (de-novo method) compared to when they were placed on patients’ front (the usual method) during echocardiography. To the best of our knowledge, this comparison in the placement of the leads for ECG monitoring during echocardiography was performed for the first time. When electrodes were on the front side, the P wave and QRS wave amplitudes between the red and green electrodes were greater than the amplitudes measured when the electrodes were placed on the back. However, in our experiences, the P wave amplitudes on the back side were sufficient to detect the cardiac cycle.

When performing echocardiography, P and QRS waves are simultaneously recorded [2]. Thus far, appropriate electrode positions during echocardiography have not been clearly determined, and have depended on the examiner conducting the test. Based on the concept of Einthoven’s triangle, a three-point electrode position is used throughout the world, where the P wave amplitude is the most visible in the II lead (red-green) because the axis of the heart is almost in the same direction as the lead. When the green electrode is attached to the left lateral abdomen on patients’ front side, the code between the electrode and the machine sometimes interferes with the performance of echocardiography. In addition, when the green electrode is attached to a leg, a longer code is required and it sometimes interferes with the performance of the echocardiography. When echocardiography is carried out in patients with thick hair, burns, or skin lesions on their front sides, front-side monitoring is difficult to accomplish during echocardiography. In such cases, back-side monitoring is considered useful.

When comparing the red and green electrodes on the front and back positions, attachment on the back gave rise to smaller BF-breath values than when attached to the front side. Respiration might cause BF-breaths and detachment of monitor seals, and subsequently, a noisy baseline might appear on the monitor. Furthermore, the back-side positioning provided a larger P wave amplitude divided by BF-breath than the front side did. The main respiratory muscles include the diaphragm and intercostal muscles [6]. The intercostal muscles are attached to the thoracic vertebrae as the hinge of the respiratory motion; thus, the front side physically moves greater than the back. Thus, back-side monitoring has less influence on respiratory movements than front-side monitoring does, and, hence, back-side monitoring could be a more appropriate method when performing echocardiography in patients with harsh respiration rates. We sometimes experience ECG monitor changes that are due to respiration, which can make it difficult to detect P waves. To date, no studies have evaluated the effects of respiration on ECG monitoring. The detection of P waves is important to confirm a patient’s diastolic phases, such as their E and A waves on echocardiography. When we see patients with vague P waves on echocardiography, back-side monitoring is one of the options that can help prevent misdiagnosis.

Back-side monitoring is possible when patients are in positions other than the left lateral decubitus position. The right lateral decubitus position for performing echocardiography is important for the diagnosis of patients with partial absence of the pericardium [7,8]. One patient with aortic stenosis in our study moved smoothly from the left to the right decubitus position using the back-side monitor, when we measured the peak velocity of the aortic valve flow from the right parasternal view. The echocardiography guideline states that the inferior vena cava should be measured in the supine position [2]. In this study, we effortlessly conducted echocardiography on all patients while in a supine position.

Methods for estimating respiratory rate from ECG signals based on variations of the QRS complex itself have been highlighted [9-11]. However, in this scenario, the fluctuation of the baseline can lead to miscounting of the respiratory rate. ECG monitors need to avoid the fluctuation of the baseline in order to count the respiration rate evaluated by the QRS wave. Thus, a back-side monitor should be used instead of a front-side one. In patients with severe heart failure, early signs of worsening heart failure are important. The change in respiratory rate is detected in the early phase during echocardiography in emergency rooms [12,13]. Therefore, the back-side monitor with respiratory rate counting may be the first choice in patients with severe heart failure. 

In this paper, we limited the study to patients in echocardiography rooms; however, future research should investigate patients with severe heart diseases in emergency rooms.

## Conclusions

Attaching ECG electrodes to a patient’s back instead of their front can lead to a clearer P wave, especially during respiration. Back-side monitoring has thus emerged as an alternative approach for performing echocardiography in patients with small P waves and harsh breathing. This method helps overcome the challenges of interpreting the cardiac cycle on ECG, such as baseline fluctuations and electrode detachments, that are encountered when electrodes are placed on the patient's front side.
